# Measuring Population-Level Adolescent Mental Health Using a Single-Item Indicator of Experiences of Sadness and Hopelessness: Cross-Sectional Study

**DOI:** 10.2196/54288

**Published:** 2024-07-26

**Authors:** Jorge Verlenden, Sanjana Pampati, Melissa Heim Viox, Nancy Brener, Laima Licitis, Patricia Dittus, Kathleen Ethier

**Affiliations:** 1 Division of Adolescent and School Health National Center for Chronic Disease Prevention and Health Promotion Centers for Disease Control and Prevention Atlanta, GA United States; 2 NORC University of Chicago Chicago, IL United States; 3 Oak Ridge Institute for Science and Education Oak Ridge, TN United States

**Keywords:** adolescents, mental health, surveillance, teens, sadness, hopelessness

## Abstract

**Background:**

Population-level monitoring of adolescent mental health is a critical public health activity used to help define local, state, and federal priorities. The Youth Risk Behavior Surveillance System includes a single-item measure of experiences of sadness or hopelessness as an indicator of risk to mental health. In 2021, 42% of high school students reported having felt sad or hopeless for 2 weeks or more during the past 12 months. The high prevalence of US high school students with this experience has been highlighted in recent studies and media reports.

**Objective:**

This study seeks to examine associations between this single-item measure of experiences of sadness or hopelessness with other indicators of poor mental health including frequent mental distress and depressive symptoms.

**Methods:**

We analyzed survey data from a national sample of 737 adolescents aged 15-19 years as a part of the Teen and Parent Surveys of Health. Participants were recruited from AmeriSpeak, a probability-based panel designed to be representative of the US household population. Feeling sad or hopeless was operationalized as a “yes” response to the item, “During the past 12 months, did you ever feel so sad or hopeless almost every day for 2 weeks or more in a row that you stopped doing some usual activities?” Unadjusted and adjusted prevalence ratios (aPRs) were calculated to examine associations between the single-item measure of having felt sad or hopeless almost every day for 2 weeks with moderate to severe depressive symptoms, frequent mental distress, and functional limitation due to poor mental health. Adjusted models controlled for age, race and ethnicity, sex assigned at birth, and sexual identity.

**Results:**

Overall, 17.3% (unweighted: 138/735) of adolescents reported that they felt sad or hopeless for 2 weeks or more during the past 12 months, 30.2% (unweighted: 204/716) reported moderate to severe depressive symptoms, 18.4% (unweighted: 126/732) reported frequent mental distress, and 15.4% (unweighted: 107/735) reported functional limitation due to poor mental health. After adjusting for demographics, adolescents who reported that they felt sad or hopeless for 2 weeks or more were 3.3 times as likely to report moderate to severe depressive symptoms (aPR 3.28, 95% CI 2.39-4.50), 4.8 times as likely to indicate frequent mental distress (aPR 4.75, 95% CI 2.92-7.74), and 7.8 times as likely to indicate mental health usually or always interfered with their ability to do things (aPR 7.78, 95% CI 4.88-12.41).

**Conclusions:**

Associations between having felt sad or hopeless for 2 weeks or more and moderate to severe depressive symptoms, frequent mental distress, and functional limitation due to poor mental health suggest the single-item indicator may represent relevant symptoms associated with poor mental health and be associated with unmet health needs. Findings suggest the single-item indicator provides a population-level snapshot of adolescent experiences of poor mental health.

## Introduction

Population-level monitoring of adolescent mental health is a critical public health activity [1]. Data from health surveillance systems of children and adolescents are used to track mental health trends, identify factors that influence mental health outcomes, and inform public health–related decision-making [2]. National surveillance systems, such as the Youth Risk Behavior Surveillance System (YRBSS), National Survey of Children’s Health, and the National Survey on Drug Use and Health, include various measures of child and adolescent mental health. No single surveillance system captures all aspects of adolescent mental health (eg, disorders, mental health concerns, or suicide), and no single indicator can summarize the many dimensions of mental health [3]. However, by harmonizing across data systems and furthering our understanding of what each data system and measure captures, a more complete picture of adolescent mental health can be established. Indeed, a number of studies have evaluated mental health measures in public health surveillance systems through descriptive comparisons of responses across items and data systems [3-5].

One such measure of poor adolescent mental health that has received growing attention in recent studies and media reports is a single-item measure of experiences of sadness or hopelessness [6,7]. Since 1999, the Centers for Disease Control and Prevention’s (CDC) YRBSS has included this single-item measure [8]. In 2021, 42% of high school students reported having felt sad or hopeless for 2 weeks or more during the past 12 months, and the prevalence of this experience significantly increased from 2011 to 2021 [9]. Further, disparities in this experience by sex, race/ethnicity, sexual identity, and sex of sexual contacts have been documented [9].

This descriptive study seeks to expand the understanding of this single-item measure by examining its alignment with established measures of poor mental health.

## Methods

### Overview

We analyzed survey data from a national sample of 737 adolescents aged 15-19 years. Data were collected by NORC at the University of Chicago, May to September 2022, as part of the Teen and Parent Surveys of Health (TAPS). Participants were recruited from AmeriSpeak, a probability-based panel designed to be representative of the US household population. Selected US households are sampled using area probability and address-based sampling from the NORC National Sample Frame to form the AmeriSpeak general adult panel. Recruitment occurs by US mail, telephone, and field interviews. In addition to the general adult panel, there are also subpanels of specific populations, including the AmeriSpeak teen panel of adolescents aged 13-17 years [7]. Adolescent TAPS participants were recruited in three ways. First, adolescents aged 18-19 years were directly recruited from the AmeriSpeak general adult panel. Second, adolescents aged 15-17 years were recruited from the AmeriSpeak teen panel. Third, we identified panelists who were parents of adolescents aged 15-17 who could consent for their child to participate in TAPS, even if they were not members of the AmeriSpeak teen panel. Panel recruitment and methodology have been published elsewhere [7,8].

### Ethical Considerations

Participants received the equivalent of US $20 in AmeriPoints, redeemable for a gift card [7,8]. Teens aged 15-17 years required parental consent to participate. Parents also received the equivalent of US $2 in AmeriPoints for completing the screener and consent survey. This activity was reviewed by the CDC and conducted consistent with applicable federal law and CDC policy; the study was also reviewed and approved by NORC’s Institutional Review Board (IRB protocol 20.07.10, project 8644). The survey was conducted confidentially.

### Measures

The analysis included four indicators of poor mental health: (1) felt sad or hopeless, based on a *Yes* response to the YRBSS item, “During the past 12 months, did you ever feel so sad or hopeless almost every day for 2 weeks or more in a row that you stopped doing some usual activities?”; (2) depressive symptoms, measured using the 8-item Patient Health Questionnaire (PHQ-8) for adolescents using a cutoff score of ≥10 to indicate moderate to severe depressive symptoms versus none, minimal, or mild [9,10]; (3) frequent mental distress, dichotomized as always or most of the time versus sometimes, rarely, or never for the question, “During the past 30 days, how often was your mental health not good? (Mental health includes stress, anxiety, and depression)”; and (4) functional limitation due to poor mental health, based on a response of always or usually versus sometimes, rarely, or never to the question, “During the past 12 months, how often has your mental health interfered with your ability to do things other young people your age do?” The PHQ-8 is established as a valid diagnostic and severity measure of depressive symptoms and is used in clinical and population studies to assess symptoms indicative of depressive disorders [9-12]. Items 3 and 4 were derived from a health-related quality of life measure and have demonstrated validity and reliability for population health surveillance [13,14].

### Analysis

The following demographic characteristics were examined: sex assigned at birth, age, race/ethnicity, and sexual identity. We present the weighted prevalence of each indicator of poor mental health overall and by demographic characteristics. Missing observations were excluded in calculations for each variable. To examine associations between feeling sad or hopeless and the other indicators of poor mental health, we used predicted margins in logistic regression to calculate prevalence ratios and adjusted prevalence ratios (aPRs), adjusting for demographics and accounting for the complex survey design.

## Results

Overall, 17.3% (unweighted: 138/735) of adolescents reported feeling sad or hopeless for 2 weeks or more during the past 12 months, 30.2% (unweighted: 204/716) reported moderate to severe depressive symptoms, 18.4% (unweighted: 126/732) reported frequent mental distress, and 15.4% (unweighted: 107/735) reported functional limitation due to poor mental health (Table 1).

Adolescents reporting that they had felt sad or hopeless had 3.3 times the prevalence of moderate to severe depressive symptoms (aPR 3.28, 95% CI 2.39-4.50), 4.8 times the prevalence of frequent mental distress (aPR 4.75, 95% CI 2.92-7.74), and 7.8 times the prevalence of functional limitation due to poor mental health (aPR 7.78, 95% CI 4.88-12.41) compared to those not reporting sadness or hopelessness (Table 2).

**Table 1:** Percent distribution of demographic characteristics of adolescent respondents by having felt sad or hopeless almost every day for 2 weeks (past 12 months), moderate to severe depressive symptoms (past 2 weeks), frequent mental distress (past 30 days), and functional limitation due to poor mental health usually or always (past 12 months)^a^.

		Overall, n	Felt sad or hopeless almost every day for 2 weeks (past 12 months)^b^, n (%)	Moderate to severe depressive symptoms, PHQ-8^c^ score ≥10 (past 2 weeks)^d^, n (%)	Frequent mental distress (past 30 days)^e^, n (%)	Functional limitation due to poor mental health *usually or always* (past 12 months)^f^, n (%)
Total		737	138 (17.3)	204 (30.2)	126 (18.4)	107 (15.4)
**Sex assigned at birth^g^**						
	Female	392	96 (20.8)	138 (38.5)	87 (22.8)	75 (19.4)
	Male	337	39 (13.5)	63 (21.4)	39 (14.5)	32 (11.8)
**Age (years)**						
	15-17	529	93 (15.1)	123 (24.4)	77 (14.0)	64 (13.1)
	18-19	208	45 (21.1)	81 (39.6)	49 (25.8)	43 (19.2)
**Race/ethnicity^h^**						
	White, non-Hispanic	372	64 (15.5)	100 (29.8)	55 (15.5)	52 (15.9)
	Black, non-Hispanic	123	21 (18.4)	30 (23.0)	19 (20.4)	15 (14.9)
	Hispanic/Latino	153	36 (23.5)	53 (36.3)	35 (25.4)	25 (17.4)
	All other races, non-Hispanic	89	17 (9.7)	21 (26.8)	17 (13.5)	15 (8.9)
**Sexual identity^i^**						
	Lesbian/gay	29	11 (36.5)	13 (41.2)	7 (18.4)	10 (31.1)
	Straight/heterosexual	561	77 (13.7)	124 (26.6)	79 (17.4)	61 (13.4)
	Bisexual	75	26 (34.0)	36 (53.2)	20 (27.1)	16 (22.5)
	Another identity/unsure	72	24 (21.1)	31 (32.7)	20 (17.4)	20 (18.7)

^a^Table shows unweighted numbers (n) and weighted percentages (%). Missing observations were excluded in the calculation of percentages for each variable.

^b^Felt sad or hopeless is based on a “Yes” response to the question “During the past 12 months, did you ever feel so sad or hopeless almost every day for 2 weeks or more in a row that you stopped doing some usual activities?”

^c^PHQ-8: 8-item Patient Health Questionnaire.

^d^Having moderate to severe depressive symptoms is based on a sum score of ≥10 on the PHQ-8 and aligns with depressive symptom severity cutoff guidelines for the PHQ, measuring number and frequency of symptoms experienced in the past 14 days.

^e^Frequent mental distress is based on a response of “Always” or “Most of the time” to the question “During the past 30 days, how often was your mental health not good? (Mental health includes stress, anxiety, and depression).” Response options were “Always,” “Most of the time,” “Sometimes,” “Rarely,” and “Never.”

^f^Functional limitation due to poor mental health is based on response of “Always” or “Usually” to the question “During the past 12 months, how often has your mental health interfered with your ability to do things other young people your age do?” Response options were “Always,” “Usually,” “Sometimes,” “Rarely,” or “Never.”

^g^Adolescents reported sex at birth in response to the survey question: “What sex were you assigned at birth, on your original birth certificate?”

^h^All other races category included American Indian, Alaska Native, Native Hawaiian or Pacific Islander, some other race, or selected more than one race category.

^i^Sexual identity was based on response to the question “Which of the following best represents yourself?” Adolescents selected from the following response options: “Lesbian or gay”; “Straight, that is, not lesbian or gay”; “Bisexual”; “Something else”; “I don’t know the answer.” Responses of “Something else” or “I don’t know the answer” were coded as “Another identity/Unsure.”

**Table 2:** Associations between adolescent report of having felt sad or hopeless almost every day for 2 weeks (past 12 months) and moderate to severe depressive symptoms (past 2 weeks), frequent mental distress (past 30 days), and functional limitation due to poor mental health usually or always (past 12 months).

		Moderate to severe depressive symptoms (PHQ-8^a^ score ≥10; past 2 weeks)^b^				Frequent mental distress (past 30 days)^c^				Functional limitation due to poor mental health usually or always (past 12 months)^d^			
		Weighted percentage (%)^e^		PR^f^ (95% CI)	aPR^g^ (95% CI)^h^	Weighted percentage (%)		PR (95% CI)	aPR (95% CI)	Weighted percentage (%)		PR (95% CI)	aPR (95% CI)
		Yes	No			Yes	No			Yes	No		
**Felt sad or hopeless almost every day for two weeks, past 12 months^i^**													
	Yes	74.5	25.5	3.59 (2.65-4.86)	3.28 (2.39-4.50)	55.0	45.0	5.05 (3.11-8.18)	4.75 (2.92-7.74)	56.0	44.0	8.02 (4.93-13.04)	7.78 (4.88-12.41)
	No	20.7	79.3	Reference	Reference	10.9	89.1	Reference	Reference	7.0	93.0	Reference	Reference

^a^PHQ-8: 8-item Patient Health Quetionnaire.

^b^Having moderate to severe depressive symptoms is based on a sum score of ≥10 on the PHQ-8 and aligns with depressive symptom severity cutoff guidelines for the PHQ, measuring number and frequency of symptoms experienced in the past 14 days.

^c^Frequent mental distress is based on a response of “Always” or “Most of the time” to the question “During the past 30 days, how often was your mental health not good? (Mental health includes stress, anxiety, and depression).” Response options were “Always,” “Most of the time,” “Sometimes,” “Rarely,” and “Never.”

^d^Functional limitation due to poor mental health is based on response of “Always” or “Usually” to the question “During the past 12 months, how often has your mental health interfered with your ability to do things other young people your age do?” Response options were “Always,” “Usually,” “Sometimes,” “Rarely,” or “Never.”

^e^Table shows weighted row percentages (%). Missing observations were excluded in calculations for each variable.

^f^PR: prevalence ratio.

^g^aPR: adjusted prevalence ratio.

^h^Adjusted for age, race and ethnicity, sex assigned at birth, and sexual identity.

^i^Felt sad or hopeless is based on a “Yes” response to the question “During the past 12 months, did you ever feel so sad or hopeless almost every day for 2 weeks or more in a row that you stopped doing some usual activities?”

## Discussion

Our study identified strong associations between having felt sad or hopeless and having moderate to severe depressive symptoms, frequent mental distress, and functional limitation due to poor mental health. Results suggest the single-item YRBSS measure offers a depiction of poor mental health among adolescents and support its use in population studies.

Mental health is a broad concept that includes a range of functioning across various contexts and environments [3]. Clinical mental health assessments, especially for children and adolescents, often involve a multicomponent process that includes structured tests and interviews with multiple informants [9,15]. However, use of single-item indicators or concise item sets is essential to reducing respondent burden in the collection of public health surveillance data [3-5]. Strengths of the YRBSS measure include its brevity and plain language, along with its association with other indicators of poor mental health that suggest unmet mental health needs, which together support its utility as a population-level measure for adolescents. Future research might examine associations between the YRBSS item and other mental health measures as well as compare responses by demographic characteristics.

Several limitations of our study must be acknowledged. First, we used data from a small sample of adolescents recruited from a panel, and we found prevalence of having felt sad or hopeless among this sample to be far lower than the national prevalence found in the 2021 YRBSS cycle [8,9]. Although data were weighted to represent the US household population, generalizability to the US adolescent population may be limited. Second, because the survey was administered in English, experiences of teens with limited English proficiency may not be represented. Third, parental consent was required for teens aged 15-17 years to participate, which may introduce selection bias. Fourth, recall periods varied for the sets of questions comprising the mental health indicators thereby limiting our ability to compare experiences within the same time frame.

Population-level monitoring of adolescent health behaviors is a critical public health activity that helps ensure awareness of changing needs of adolescents and helps support priority-setting for public health programming [1-3,16]. The associations between having felt sad or hopeless for 2 weeks or more and moderate to severe depressive symptoms, frequent mental distress, and functional limitation due to poor mental health suggest the single-item indicator may represent relevant symptoms associated with poor mental health and be associated with unmet health needs. Findings from this study support the value of this item for use in population studies of adolescents and can enhance understanding of results of the YRBSS moving forward.

## Abbreviations

aPR: adjusted prevalence ratio
CDC: Centers For Disease Control and Prevention
PHQ-8: 8-item Patient Health Questionnaire
TAPS: Teen and Parent Surveys of Health
YRBSS: Youth Risk Behavior Surveillance System
